# Connectivity Analysis for Multivariate Time Series: Correlation vs. Causality

**DOI:** 10.3390/e23121570

**Published:** 2021-11-25

**Authors:** Angeliki Papana

**Affiliations:** Department of Economics, University of Macedonia, 54636 Thessaloniki, Greece; angeliki.papana@gmail.com or apapana@uom.edu.gr

**Keywords:** connectivity, correlation, causality, dependencies, lagged, instantaneous, complex networks

## Abstract

The study of the interdependence relationships of the variables of an examined system is of great importance and remains a challenging task. There are two distinct cases of interdependence. In the first case, the variables evolve in synchrony, connections are undirected and the connectivity is examined based on symmetric measures, such as correlation. In the second case, a variable drives another one and they are connected with a causal relationship. Therefore, directed connections entail the determination of the interrelationships based on causality measures. The main open question that arises is the following: can symmetric correlation measures or directional causality measures be applied to infer the connectivity network of an examined system? Using simulations, we demonstrate the performance of different connectivity measures in case of contemporaneous or/and temporal dependencies. Results suggest the sensitivity of correlation measures when temporal dependencies exist in the data. On the other hand, causality measures do not spuriously indicate causal effects when data present only contemporaneous dependencies. Finally, the necessity of introducing effective instantaneous causality measures is highlighted since they are able to handle both contemporaneous and causal effects at the same time. Results based on instantaneous causality measures are promising; however, further investigation is required in order to achieve an overall satisfactory performance.

## 1. Introduction

There are various challenges in the analysis of multivariate high-dimensional systems, such as in the analysis of financial and neurophysiological data. The goal of each application and the features of the examined data should be considered in order to determine the suitable connectivity analysis scheme. For example, financial time series are nonstationary, contain nonlinearities and exhibit volatility clustering, whereas data in neuroscience experiments may present a high temporal resolution, be subject to artifacts, periodic respiratory or cardiac noise.

Connectivity analysis focuses on identifying the interdependence relationships of the variables of a complex system. Connectivity measures can be subdivided into two main categories based on whether they quantify the direction of a relationship. Nondirectional measures assume that variables evolve in synchrony and the connectivity is examined based on symmetric measures, such as dependence measures. On the other hand, directed measures quantify the causal effects among the variables, assuming that causes precede their effects in time, such as Granger causality measures [1]. A further subdivision of both categories differentiates between model-based and model-free connectivity measures. Both categories of measures consist of measures calculated in the time, frequency or phase domain. Spectral measures of dependence infer the dependence between oscillatory components of the examined data.

Since there is an abundance of connectivity measures that have been developed so far, there is also an urgent need to compare them and clarify the usefulness of each method. Comparisons are mainly performed in terms of applications of interest. Indicatively, comparisons of correlation measures can be found in [2,3,4,5,6,7,8,9,10,11], among synchronization measures in [12,13,14,15,16,17] and among causality measures in [16,18,19,20,21,22,23,24,25,26]. However, the bibliography lacks comprehensive connectivity evaluations, including both correlation and causality methods. Causality is either solely utilized or supplementary to correlation, i.e., correlation is a sign of causality, although it is not sufficient to infer causality [27,28,29,30].

There are a number of limitations and common pitfalls regarding the connectivity measures that complicate the correct inference of the connectivity of a system. For example, correlation measures are usually assuming linearity and do not handle outliers, are affected by the sample size and there are also interpretational mistakes when discussing their outcomes [31,32,33,34,35]. Some limitations relevant to causal measures are the sample size bias, the common input problem, the effect of noise and the curse of dimensionality [36,37,38,39,40,41,42]. We will briefly discuss these problems below.

The purpose of this paper is to provide a brief review on connectivity analysis, enlisting the most well known connectivity measures and identifying the possible pitfalls when forming connectivity networks. Selecting between correlation and causality measures for inferring the connectivity structure of an examined system is of the key interest of this study; therefore, it is essential to demonstrate the performance and pitfalls of the different connectivity measures focusing on those that have not been stressed and investigated extensively so far. In particular, we examine the influence of the existence of contemporaneous interdependencies to the extracted causal network, the effect of causal relationships when forming correlation networks and finally, inquire the case of having both contemporaneous and causal relationships among the variables of a multivariate system.

## 2. Non-Directional Connectivity Measures

The examination of the dependencies among the variables of a complex system is an essential task in statistics. Non-directional, symmetric connectivity measures aim to capture and quantify the strength of associations between the variables of a complex system, without indicating the direction of the relationship. Correlation measures are utilized for the characterization of statistical relationships.

The most commonly used correlation measure is the Pearson correlation coefficient, also known as the Pearson product-moment correlation coefficient, or bivariate correlation or correlation coefficient [43]. It is expressed as a normalised measurement of the covariance and it can only account for the linear relationships of the variables. It is not robust in case of outliers and is sensitive to the data distribution.

Many alternatives to the correlation coefficient have been developed, aiming to address its limitations. Rank-based analogues of the correlation coefficient do no make any assumptions about the frequency distribution of the variables and do not assume that a linear relationship between the variables exists, whereas they can be used both for variables measured on interval scales but also at ordinal levels. Spearman’s rank correlation coefficient [44] and Kendall’s rank correlation coefficient [45] are the most common non-parametric correlation alternatives to the correlation coefficient.

Partial correlation measures quantify the degree of association between two variables while controlling for the effect of any additional variables of the examined system. Their main contribution is that they can uncover spurious relationships and detect hidden relationships. The most common partial measure is the linear partial correlation coefficient, which can be estimated based on the bivariate linear correlation coefficients of the examined variables. Its nonparametric analogue is the partial Spearman’s rank correlation coefficient.

Hoeffding’s test of independence is a test of correlation for two variables with continuous distribution functions [46]. Multivariate Hoeffding’s phi-squared test is an extension of Hoeffding’s test of independence that expressed the multivariate association [47]. Biweight midcorrelation is a measure of similarity between samples. It is median-based and, therefore, is more robust to outliers than Pearson’s correlation.

The coefficient of determination is based on linear regression models [48] and is equal to the squared value of the correlation coefficient. Pearson’s correlation can be zero for dependent random variables. Distance correlation was developed aiming to face this limitation, i.e., a zero distance correlation implies independence [49,50]. Later, partial distance correlation was developed with methods for dissimilarities [51].

The odds ratio quantifies the dependence between two binary variables and ranges from zero to infinity. Yule [52,53] introduced two normalized versions of the odds ratio, namely the Yule’s *Q* and the coefficient of colligation or Yule’s *Y*, respectively. Various different extensions have been defined subsequently, such as Digby’s coefficient *H* [54] and coefficient Y* [55].

Copulas are used to model the dependence between random variables. For example, the Randomized Dependence Coefficient measures the dependence between multivariate random variables [56]. Copula correlation is a copula-based measure exploiting kernel density estimators [57]. A class of copula-based dependence coefficients have been developed, which are computationally efficient and can handle a wide range of associations [58,59,60,61,62,63].

The continuous analysis of variance test (CANOVA) is a nonlinear correlation measure defined by the neighborhoods of the data points of two continuous variables [64]. The rationale of CANOVA is that neighboring values of the first variable should lead to correlation of neighboring values of the second variable.

An ensemble of information-theoretic alternatives to the linear correlation coefficient aim to account for nonlinearities and temporal dependencies in the data. Mutual information is used as a generalized correlation measure that quantifies nonlinear associations [65,66]. Mutual information is also considered to form the Mutual Information Matrix for the study of nonlinear interactions in multivariate time series [67]. Time-delayed mutual information takes into account the lag difference of the variables [65].

Nonlinear correlation information entropy is specified by the rank sequences that are obtained from the original data sets [64]. The entropy correlation coefficient [68] and the entropy coefficient of determination [69] were utilized due to their desirable properties, such as being easily explicable and able to express nonlinear associations. Maximal information coefficient has been utilized for the detection of linear and non-linear relationships in large data sets [70]. The partial maximal information coefficient captures the association between two variables, removing the effect of a third random variable [71]. The time-delayed mutual information of the phase has been introduced for determining nonlinear synchronization in electrophysiological data [72].

Time domain measures express the variation of amplitude of signal with time. Frequency domain analysis of signals is performed in reference to frequency, rather than time. Coherence function quantifies linear correlations in the frequency domain [73]. Cross-coherence is the equivalent measure of cross-correlation in the frequency domain [74]. Partial coherence has been defined in order to quantify the strength of a conditional relationship between two neurons and is suitable for multivariate analysis by using the predictor to distinguish direct connection from common inputs [75]. As an extension, multivariate partial coherence analysis has been introduced [76].

Phase synchronization stems from the notion of synchronization of chaotic oscillators, whereas the global diversion of the phases of the signals is examined [77,78]. Different measures of phase synchronization have been developed, such as the mean phase coherence [12,79].

Phase locking is a key notion in dynamical systems. Phase locking value is a measure robust to fluctuations in amplitude that quantifies the absolute value of the mean phase difference between two signals [12,78].

Recurrence analysis examines how close the states of a dynamical system are after some time [80]. Its extension includes the cross recurrence plots, which are the bivariate extension that examines the dependencies between two different systems [81,82]. Several measures stem from recurrence analysis, such as recurrence rate, determinism and maximal length of diagonal structures [83,84].

We should note here that most correlation measures are bivariate since extensions of them in the multivariate case most probably lead to directional measures, such as the conditional mutual information, which is an extension of mutual information that accounts for the remaining variables of the system which infers about the directionality of the information flow [85,86,87].

A list of some well known correlation measures in time and frequency domain is displayed in Table 1.

The different correlation measures have been vastly applied in different fields, such as in finance, neurophysiology, meteorology, biology and engineering. Applications include the identification of genomic associations [89,90], the examination of the association of proteins in the pathogenesis of Parkinson’s disease [91], noise reduction [92,93], identification of disease-specific biomarker genes [94], multimodal image registration [95], portfolio optimization [96,97], investment decisions [98], wind power combination prediction [99], genetic interactions [100], artificial neural network model development that concerns water treatment plants [101], testing tourism economies and islands’ resilience to the global financial crisis [102], electroencephalograms (EEG) analysis [103], the study of financial markets [104,105], recognizing multiple positive emotions by analyzing brain activities [106], identifying meteorological parameters that play a major role in the transmission of infectious diseases such as COVID-19 [107] and stock trend prediction [108,109].

## 3. Directional Connectivity Measures

Directional connectivity measures seek to infer the direction of the relationship from the data samples, relying on the principle that causes precede their effects. The most common procedure of causal discovery is Granger causality, where probabilistic causation relies on the concept that causes change the probabilities of their effects [1,110].

Model-based directional approaches assume the linearity of interactions. The standard linear Granger causality is the pioneer technique based on autoregressive models that seeks to determine whether prediction of the target (driven) variable can be improved by exploiting past values of the source (driving) variable [1].

Various model-based extensions of the standard Granger causality test have been developed so far. The conditional Granger causality is its multivariate extension that exploits all the available information of the observed data [111]. Partial Granger causality is an extension of conditional Granger causality developed to face the problem of exogenous inputs and latent variables [112]. Further parametric causality methods have been introduced, such as methods defined on radial basis functions [113], kernel functions [114] and nonlinear autoregressive exogenous models [115].

Non-parametric extensions of Granger causality to nonlinear cases in the time domain include the Baek and Brok test [116], the Hiemstra and Jones test [117] and the Diks and Panchenko test [118], and [119] extend the Hiemstra and Jones test in multivariate settings.

Numerous directional measures stem from information theory. These model-free approaches infer linear but also nonlinear interactions. Transfer entropy is the most well-known information measure for studying directed interactions [120]. Partial transfer entropy extends the bivariate transfer entropy to the multivariate case, where confounding variables are also considered in the estimations [121,122]. Some further information causality measures based on the nonuniform embedding scheme are the partial transfer entropy [123], (partial) mutual information on mixed embedding [124,125] and transfer entropy based on low-dimensional approximations of conditional mutual information [126,127].

Linear cross-correlation is the simplest and most well known synchronization measure defined as the ratio of covariance to root-mean variance of the two signals. Event synchronization is another simple and computationally efficient method that quantifies synchronicity and time delay patterns between signals [128].

Various nonlinear interdependence measures have been developed in regards of nonlinear prediction theory that use the neighborhoods of the reconstructed points of the state space aiming to determine the nonlinear driver-response relationships [129,130,131,132,133,134]. As an extension of the above, the (conditional) extended Granger causality further employs a linear model for all the points in the neighborhood of each reference point of the reconstructed state space [135]. A more recent bivariate causality method based on nonlinear state space reconstruction can be found in [136]. Empirical dynamic modeling (convergent cross mapping) is utilized for the definition of this measure. It has been introduced to inferring causality from complex systems that do not satisfy the separability assumption, i.e., when the cause and the effect are non-separable.

Graphical models have been suggested by [137] to account for probabilistic independence relationships between variables without relying on temporal information. Probabilistic graphical models are a combination of graph theory and the probability theory.

The field of causal discovery was signified by [138,139], where causal interpretation of the graphs was succeeded based on Bayesian network models. The PC algorithm is the pioneer structure-learning algorithm for directed graphs [138] under the assumption of the Causal Markov condition. After the introduction of the PC algorithm and the Fast Causal Inference [140], an ensemble of different causal discovery methods based on graphical models has been developed [141,142,143,144]. Markov discovery algorithms such as the PC algorithm cannot be directly used for the time series. Therefore, ref. [145] adapted the Fast Causal Inference algorithm for time series.

The Peter Clark momentary conditional independence algorithm is a causal discovery method that incorporates linear or nonlinear conditional independence tests to determine the causal networks from multivariate time series data [146]. This measure is designed for climate applications; therefore, it can handle strong interdependencies in the sample. An extension of this measure aiming to improve the computational efficiency is the Fast Approximate Causal Discovery Algorithm [147].

Data from time domain can be converted to the frequency domains with mathematical operators, such as the Fourier transform, which converts a time function into a sum or integral of sine waves of different frequencies. Causality measures from the frequency domain have been widely applied for the analysis of neurophysiological data. The majority of the developed spectral measures are based on linear models, and thus can only detect linear causal effects in the frequency domain, such as Geweke’s spectral Granger causality [111], the directed transfer function [148], the partial directed coherence [149], the direct Directed Transfer Function [150], the Generalized Partial Directed Coherence [151], the Phase Slope Index [152]. Recently, a frequency-domain approach for testing for short-and long-run causality has been introduced in [153].

Nonparametric methods have been also employed, such as the nonparametric approach based on Fourier and wavelet transforms in [154], the nonparametric partial directed coherence [155] and the DEKF-based extension of partial directed coherence, where the parameters of the time-varying autoregressive model are estimated using the Dual Extended Kalman Filter (DEKF) [156]. Further, the nonlinear partial directed coherence aims to model the nonlinear relationships of the examined time series using nonlinear models and generalized frequency response functions [157].

Granger causality relationships are examined by considering the past values of the involved variables. However, the prediction of the target variable, may at cases be improved by including the available current information of the source variable. In such a case, the instantaneous causality relation between the source and target variable should be considered [158]. For example, contemporaneous relationships are present if the regression residues of the data are correlated.

Within the framework of stationary autoregressive modeling, the instantaneous causality is usually tested by using Wald tests for zero restrictions on the innovation’s covariance matrix. Extended Granger causality accounting for zero-lag effects in the linear regression schemes implemented by the VAR model [159]. Instantaneous causality in presence of non constant unconditional variance is examined in [160]. Instantaneous causality measures defined on structural vector causal models are presented in [161,162,163,164].

A causality framework in frequency domain that considers instantaneous effects is introduced in [165]. An instantaneous measure of causality which is relying on the information versions of directed transfer entropy and partial directed coherence estimated after decomposing the coherencies and partial coherencies is presented in [166]. Instantaneous Granger causality measures based on the the Hilbert-Huang transform are introduced in [167].

Compensated transfer entropy is a nonlinear causality measure that regards contemporaneous relationships [168,169]. A multivariate Granger causality measure including instantaneous variables in the conditional set based on decomposition of conditional directed information is discussed in [170]. Partial mutual information from mixed embedding that considers also zero-lag effects, denoted as PMIME0, faces the problem of determining the connectivity network from multivariate time series in the presence of unobserved variables [171]. Finally, PCMCI+ is a causality measure based on conditional independence tests that searches for causal and contemporaneous parents in order to infer lagged and contemporaneous causal relationships.

A list of well known directional connectivity measures in time and frequency domain is displayed in Table 2.

The pioneer Granger non-causality test has been developed for analyzing financial data [1]; however, it is now vastly applied in various fields, such as for the analysis of magnetoencephalography (MEG) and electroencephalography (EEG) data [173,174]. Granger causality and its extensions, along with the alternative causality measures that have been developed afterwards, are vastly used in different applications. Among others, causality measures are utilized in financial applications, e.g., for the examination of the relation of stock markets [175,176], in neuroscience, e.g., for the analysis of brain structures and physiological time series [150,169,177], in seismology, e.g., for the analysis of earthquake data [178], in geoscience, e.g., for the discovery of weather and vegetation conditions on global wildfire [179], in meteorology, e.g., for modeling the air quality [180,181], and in epidemiology [182,183].

## 4. Limitations and Pitfalls of Connectivity Measures

The estimation of symmetrical and causal relationships from observational data has been vastly explored, along with the limitation and pitfalls of the corresponding measures [35,38,184,185,186,187,188,189,190]. Naturally, depending on the examined application, different additional issues may arise that should be addressed. For example, when analyzing electroencephalogram data, spurious functional connectivity may arise due to the common reference problem, i.e., as a result from the usage of a common reference channel. Therefore, connectivity measures that are sensitive to correlations at a zero time may give erroneous indications depending on the relative strength of the potential fluctuations at the recording and reference locations.

Linear model-based connectivity measures assume linearity of the relationships [191,192], whereas outliers can strongly affect them [191,192]. At cases, relationships can be linearised, by transforming the variables, e.g., by considering a logarithmic transformation. Alternatively, for monotonic nonlinear relations, rank-based measures can be utilized. If these solutions cannot be applied, then nonparametric and nonlinear measures are more appropriate. For example, rank-based measures and information-based measures are robust to outliers.

In general, measures of connectivity are biased, and, therefore, under the null hypothesis of no connectivity the estimates will be different from zero. Accurate estimation of connectivity measures requires sufficient sample sizes [193]. Guidelines for sufficient sample sizes have been presented for different scenarios [194,195,196], whereas solutions for different applications with small samples have been proposed [197,198,199].

Real data may entail various types of noise and noise levels; there are different data measurement methods that may entail measurement errors depending on the application. For example, noise in financial data may stem from small price movements and trading noises that illustrate heavy tails. The effect of noise on correlation measures has been examined in different studies [200,201,202]. The effect of noise on Granger causality analysis has been also examined. Due to noise, erroneous causality arises and true causality is suppressed when using the standard linear Granger causality test [36]. The nonlinear causality measures are generally more stable to the effect of noise than the linear ones [26,125].

There are various reasons for inferring spurious causal effects, such as due to unobserved variables, contemporaneous relationships, common inputs, synergetic and redundant influences and strong autocorrelations in the sample. Another difficulty in causal inferring is the discrimination between direct and indirect interactions when common inputs exist, although direct causality measures have been developed for this. Robust methods that can account for latent effects of unobserved variables are an open area of investigation in connectivity analysis [203,204,205,206,207,208].

The determination of causal directionality for contemporaneous links is an emerging area. An instantaneous causal effect can be interpreted as a zero-lag causality or as a symmetric causal relationship. However, it has been noted that instantaneous causality may arise in case of common sources and latent, unobserved variables [41,171].

## 5. Correlation vs. Causality

A plethora of connectivity measures have been briefly discussed above, along with some main pitfalls and limitations. However, a key question that arises is whether to apply symmetric correlation measures or directional causality measures to infer the connectivity network of an examined system. Since connectivity of real systems is unknown, the nature of the examined data and the performance of the connectivity measures are of great importance.

Therefore, we generate synthetic time series with known connectivity structures and demonstrate the efficacy of the connectivity measures in three different scenarios. In particular, we examine the influence of different types of dependencies in the samples to the efficiency of the connectivity measures. First, we consider a simulation system with only contemporaneous dependencies and explore the performance of the connectivity measures and in particular of the causality measures. The second simulation system demonstrates the effect of time-lagged directional relationships on the connectivity measures and in particular on correlation measures that are not defined in order to detect lagged dependencies. Finally, we consider a system with contemporaneous and time-lagged directional relationships and examine the performance of the connectivity measures. To better simulate real data which are usually non-normal, the noise terms of the considered stochastic simulation systems are not exclusively Gaussian, as usually assumed in the literature, but also skewed and non-symmetrical noise terms are regarded.

Based on the equations of each simulation system, 100 realizations with sample size n=2000 are formed and different connectivity measures are computed. Specifically, we estimate the four correlation measures, four causality measures and two instantaneous causality measures. Let us examine the three-variate case, where known variables are X,Y and *Z*. The multivariate connectivity measures are similarly defined; however, instead of *Z*, an ensemble of conditioning variables Z$=Z_{1},\ldots ,Z_{K}$ exists.

The aim of the study is to provide insights on the effectiveness of the different types of connectivity measures. Therefore, an indicative selection of measures is performed since it is impossible to include the ensemble of existing connectivity measures. The examined measures cover the most commonly used types of connectivity measures.

The considered correlation measures are the following ones:

- Partial linear Pearson correlation coefficient (PPCor) $=\frac{\rho_{XY}-\rho_{XZ}\rho_{ZY}}{\sqrt{1-\rho_{XZ}^{2}}\sqrt{1-\rho_{ZY}^{2}}}$, where $\rho_{XY}=\frac{cov(X,Y)}{\sigma_{X}\sigma_{Y}}$, cov stands for covariance, and $\sigma_{X}$ and $\sigma_{Y}$ are the standard deviations of *X* and *Y*. Estimation of PPCor is performed based on “partialcorr” function from the Matlab Statistics Toolbox.
- Partial Spearman rank correlation coefficient (PSpCorr), defined similarly to PPCor but on the series of the ranks. Estimation of PSpCorr is performed based on “partialcorr” function from the Matlab Statistics Toolbox.
- Partial distance correlation (pdCor) is the extension of the distance correlation (dCor) in the multivariate case. The distance correlation of two random variables is obtained by dividing their distance covariance by the product of the distance standard deviations, i.e., $dCor\left(X,Y\right)=\frac{dCov^{2}\left(X,Y\right)}{\sqrt{dVar(X)dVar(Y)}}$. Partial distance correlation is defined based on a Hilbert space where the squared distance covariance is defined as an inner product [51]. Estimation of pdCor is performed based on R codes given in [209].
- Mutual information (MI) = H(X)−H(X|Y) can be expressed on entropy terms, where H(X) is the Shannon entropy of the variable *X*. The k-nearest neighbors (KNN) estimator has been utilized for the estimation of MI [210].

The regarded causality measures are listed below:

- Conditional Granger causality index (CGCI) is defined on the unrestricted and restricted vector autoregressive model (VAR) of order P, fitted to the time series of *X*: $CGCI_{Y\to X|Z}=\frac{lnS_{R}^{2}}{lnS_{U}^{2}}$, where the unrestricted model includes past terms from X,Y,Z variables, the restricted model omits the past terms of *X* variable and $lnS_{R}^{2}$, $lnS_{U}^{2}$ are the residual variances of the corresponding VAR models. The Matlab code for the computation of CGCI can be found in https://github.com/dkugiu/Matlab/ (accessed on 23 October 2021).
- Restricted conditional Granger causality index (RCGCI) is defined similarly to CGCI, however a modified backward-in-time selection method is used and a subset of lagged terms enter the unrestricted VAR model. Matlab codes for the computation of RCGCI can be found in https://users.auth.gr/dkugiu/ (accessed on 23 October 2021).
- Partial transfer entropy on non-uniform embedding (PTENUE) measures the direct effect of *Y* on *X* in the presence of the “appropriate” past terms of all the variables $w_{t}=\left\{w_{t}^{X},w_{t}^{Y},w_{t}^{Z}\right\}$ : $PTENUE_{Y\to X|Z}=I\left(x_{t+1};w_{t}^{Y}|w_{t}\right)$, where $x_{t+1}$ is the future value of *X* one step ahead. Matlab codes for the estimation of PTENUE can be found in http://www.lucafaes.net/its.html (accessed on 23 October 2021).
- Partial directed coherence (PDC) is based on VAR models as CGCI; however, it is defined in the frequency domain. For a frequency *f*, it is given as $PDC_{Y\to X|Z}\left(f\right)=\frac{|A_{1,2}\left(f\right)|}{\sum |A_{k,2}{\left(f\right)|}^{2}}$, where A(f) is the Fourier transform of the coefficients of the VAR model of order *P* and $A_{i,j}\left(f\right)$ is the component at the position (i,j) in the A(f) matrix. Matlab code can be provided upon request.

Finally, two instantaneous causality measures are assumed in this study:

- Partial mutual information on mixed embedding (PMIME0) is an extension of the causality measure PMIME, that also contains zero lag terms. For the estimations, the Matlab code was provided by the authors [171].
- Peter Clark momentary conditional independence algorithm (PCMCI+) addresses both lagged as well as contemporaneous causal discovery. Its an extension of PCMCI, which searches for causal parents based on conditional independence tests. The information-theoretic framework is considered here where the conditional mutual information is utilized as a general test statistic. Computations are performed using the python codes in https://github.com/jakobrunge/tigramite (accessed on 23 October 2021).

Standard free parameters and significance tests are utilized for each connectivity measure. In particular, the significance test for PPCor and PSpCorr is parametrically extracted based on the test statistic $t=\frac{r\sqrt{n-2}}{\sqrt{1-r^{2}}}$, which follows the Student’s *t*-distribution with n−2 degrees of freedom. For the estimation of MI, we consider the KNN method where k=10 neighbors. Regarding its statistical significance, it is assessed by randomly permuting the time series; *p*-values are then estimated from a one sided-test for the null hypothesis that two variables are independent. The number of permuted time series is set to be equal to 100. The significance of pdCor is also assessed using 100 permutations. As previously stated, the PDC is estimated for a range of frequencies in [0,0.5] (256 different frequencies). Significance is assessed parametrically as it is defined on VARs. The percentage of significant PDC values for each frequency is then examined. Finally, we display the percentage of significant PDC values over all frequencies and realizations, instead of displaying results for specific frequencies or frequency bands. The order of the VAR model for system 1 is set to be P=1, for system 2 we set P=3 and for system 2 we set P=3.

A parametric significance test is employed for CGCI and RCGCI since these measures are also defined on VARs [153]. Order of VAR is set as noted for PDC above. The PTENUE and PMIME0 incorporate surrogates within their estimation algorithm and no significance test is required; positive values suggest the existence of causal effects, otherwise zero values are obtained. The free parameter $L_{max}$ for the lagged terms is equal to 4 for all systems. The significance level for the test for the termination criterion for PMIME0 is 0.05, whereas for PTENUE it is set to 0.01 For both measures, we set one step ahead that the mixed embedding vector has to explain, we consider 100 surrogates for the significance test and 10 neighbors (KNN estimator). Finally, the majority rule for handling ambiguous triples is assumed and the significance level is 0.05. Finally, local permutation tests are employed within the estimation procedure of PCMCI+ to determine the causal parents.

### 5.1. Simulation System 1

**Figure 1:**
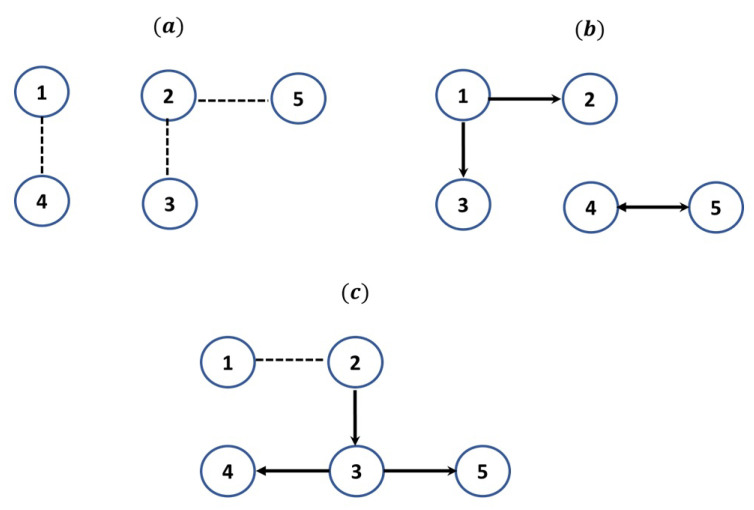
The true causal network of (**a**) S1, (**b**) S2, (**c**) S3. Dotted lines denote contemporaneous dependencies and directed arrows denote temporal (causal) dependencies.

First, we consider a five-variate stochastic nonlinear simulation system where by construction, data have known contemporaneous dependencies and there are no causal influences. The equations of the system are the following:

$$\begin{array}{ccc} x_{1t} & = & e_{1t}\\ x_{2t} & = & e_{2t}\\ x_{3t} & = & 0.8x_{2t}+e_{3t}\\ x_{4t} & = & 0.7x_{1t}\left(x_{1t}^{2}-1\right)e^{-x_{1t}^{2}/2}+e_{4t}\\ x_{5t} & = & 0.3x_{2t}+0.05x_{2t}^{2}+e_{5t} \end{array}$$

where $e_{1t}$ follows an exponential distribution with rate λ=2, $e_{2t}$ follow a chi-squared distribution with 1 degree of freedom, $e_{3t},e_{4t},e_{5t}$ follow the Gaussian distribution (mean=zero, standard deviation one) and all noise processes are independent to each other. Based on the system’s equations, significant positive linear dependencies exist between the variables $X_{2}$, $X_{3}$, whereas nonlinear ones exist between the variables $X_{1}$, $X_{4}$ and $X_{2}$, $X_{5}$ (Figure 1a).

Correlation measures are symmetrical; therefore, results are displayed by upper triangular Tables. The PPCor detects the linear correlation between $X_{2}$, $X_{3}$ and the nonlinear one between $X_{2}$ and $X_{5}$; however, the complex nonlinear relationship of $X_{1}$ and $X_{4}$ is detected with a very low percentage over the 100 realizations (Table 3). PSpCor correctly identifies the connectivity network of the system; however, it also indicates the non-direct association of $X_{3}$ and $X_{5}$ with a percentage of 100% over the 100 realizations. The pdCor and MI have similar performance with PSpCor, with MI achieving a relatively low percentage of significant correlations for the pair of variables $X_{1}$–$X_{4}$ (31%). Since MI is the only bivariate correlation measure considered, it is the only measure expected to indicate the association of $X_{1}$ and $X_{4}$. Therefore, although the system is formulated having only contemporaneous dependencies, none of the correlation measures describes the entire connectivity network of the system with complete accuracy.

On the other hand, all the direct causality measures correctly suggest that no causal effects exist among the variables of the first simulation system. RCGCI, PTENEUE, PDC and PCMCI achieve low percentages of significant causal links over the 100 realizations (around the nominal level 5%), therefore suggesting that no causal effects exist. Regarding PDC, it gives low percentages of significant effects for all the examined frequencies. No information about contemporaneous relations can be inferred from the causality measures.

Finally, the instantaneous causality measures PMIME0 and PCMCI+ are estimated. Both contemporaneous and lagged effects are extracted and reported in Table 3 for both measures. PMIME0 correctly finds the contemporaneous relationships; however, the percentage of significant relations over the 100 realizations for $X_{1}$–$X_{4}$ is relatively low (42%). Due to the estimation procedure of PMIME0, results are approximately symmetrical as long as contemporaneous effects are concerned. Regarding the lagged effects based on PMIME0, percentages of significant links are greater than the nominal level (5%) at most directions, with the highest one achieving 28% for $X_{1}$→$X_{3}$. Such a performance has been observed previously for PMIME, whereas the detection of non-coupled pairs of variables was observed with percentages larger than the considered nominal [26]; as previously mentioned, PMIME0 is the extension of the causality measure PMIME that infers about both lagged and contemporaneous dependencies. Finally, PCMCI+ correctly identifies the contemporaneous effects with high percentages over the 100 realizations. Further, low percentages for causality are obtained for all pairs of variables. The majority of the estimated percentages are slightly over the nominal level.

### 5.2. Example 2

In the second example, the causal influences between the variables are known by construction while no contemporaneous influences exist. A nonlinear vector autoregressive (VAR) model of order 3 in five variables is formed. The systems’ equations are given below:

$$\begin{array}{ccc} x_{1t} & = & 0.7x_{1t-1}+e_{1t}\\ x_{2t} & = & 0.3x_{1t-2}^{2}+e_{2t}\\ x_{3t} & = & 0.4x_{1t-3}-0.3x_{3t-2}+e_{3t}\\ x_{4t} & = & 0.7x_{4t-1}-0.3x_{5t-1}e^{-x_{5t-1}^{2}/2}+e_{4t}\\ x_{5t} & = & 0.5x_{4t-1}+0.2x_{5t-2}+e_{5t} \end{array}$$

where $e_{1t}$, $e_{5t}$ follow the Gaussian distribution (mean = zero, standard deviation=one), $e_{2t}$ follows an exponential distribution with rate λ=2, $e_{3t}$ follows beta distribution with shape parameters a=1 and b=2, $e_{4t}$ follows beta distribution with a=2 and b=1 and all noise processes are independent to each other. Based on the system’s equations, there are both nonlinear causal influences, i.e., $X_{1}$→$X_{2}$, $X_{5}$→$X_{4}$, and linear ones, i.e., $X_{1}$→$X_{3}$, $X_{4}$→$X_{5}$ (Figure 1b).

In the second simulation system, correlation measures seem to be affected by the temporal dependencies and indicate significant contemporaneous dependencies (Table 4). In particular, all the correlation measures indicate the correlated pairs of variables $X_{1}$–$X_{3}$ and $X_{4}$–$X_{5}$, whereas pdCor is also suggesting additional correlated pairs of variables ($X_{1}$–$X_{2}$, $X_{1}$–$X_{4}$ and $X_{2}$–$X_{3}$). We notice that the suggested correlated pairs of variables ($X_{1}$–$X_{3}$ and $X_{4}$–$X_{5}$) coincide with the pairs of variables with linear causal links, i.e., $X_{1}$ linearly causes $X_{3}$ and $X_{1}$ linearly causes $X_{3}$.

The linear causality measures CGCI and RCGCI infer correctly the causal relationships, however the nonlinear link $X_{1}$→$X_{2}$ achieves a relatively low percentage of significant effects over the 100 realizations (20% and 2024%, respectively). Separability assumption states that there is unique information about the target variable contained in the driving variable. When this assumption is satisfied, such as in case of linear stochastic systems, Granger causality is effective, whereas deterministic dynamical systems commonly do not satisfy the separability condition. Therefore, the ability of CGCI and RCGCI to detect nonlinear causal effects is related to the nature of the examined system and the satisfaction of the separability assumption.

The nonlinear causality measure PTENUE also correctly indicates the directional linkages; however, the nonlinear effect $X_{4}$→$X_{5}$ is detected with a percentage of 44% over the 100 realizations. PDC has the lowest performance among the causality measures. It detects $X_{1}$→$X_{3}$, $X_{4}$↔$X_{5}$ and fails to find $X_{1}$→$X_{2}$, whereas the spurious causal effects $X_{4}$→$X_{1}$ (37%), $X_{4}$→$X_{2}$ (52%), $X_{4}$→$X_{3}$ (35%) are obtained.

Regarding the instantaneous causality measures, PMIME0 does not identify contemporaneous relations, and suggests the correct causal effects. As noted in the first simulation system, the percentage of significant causal effects for the non-causal links may exceed the nominal level; however, the exported percentages are generally lower compared with those obtained for system 1. PCMCI+ performs worse than PMIME0. It suggests only temporal dependencies; however, large percentages of significant causal effects are noted for non-causal links, where the highest erroneous percentages concern $X_{2}$→$X_{3}$ (41%) and $X_{3}$→$X_{1}$ (32%); the common input variable $X_{1}$ possibly confuses PCMCI+.

### 5.3. Example 3

Finally, we consider a system with temporal and contemporaneous dependencies, i.e., lagged and zero-lag dependencies. The causal influences between the variables are known by construction. The equations of the considered system are the following ones:

$$\begin{array}{ccc} x_{1t} & = & 0.6x_{1t-2}+e_{1t}\\ x_{2t} & = & x_{1t}+0.3x_{2t-1}+e_{2t}\\ x_{3t} & = & 0.3x_{3t-1}+sin\left(x_{2t-3}\right)+e_{3t}\\ x_{4t} & = & 0.4x_{3t-2}+e_{4t}\\ x_{5t} & = & -3.2+0.5x_{3t-1}^{2}+e_{5t} \end{array}$$

where $e_{1t}$, $e_{4t}$ follow the Gaussian distribution (mean = zero, standard deviation = one), $e_{2t}$, $e_{3t}$ follow beta distribution with shape parameters a=1 and b=2, $e_{5t}$ follows gamma distribution with a=16 (shape) and b=0.25 (rate) and all noise processes are independent to each other. Based on the system’s equations, there is a contemporaneous relationship between $X_{1}$ and $X_{2}$, the linear causal influence $X_{3}$→$X_{4}$ and the nonlinear causal effects $X_{2}$→$X_{3}$, $X_{3}$→$X_{5}$ (Figure 1c).

Correlation measures correctly indicate the contemporaneous relation of $X_{1}$ and $X_{2}$, however additional relations are suggested for the pairs of variables with causal relations but also for many non causal pairs of variables (Table 5). Therefore, as already noted in the first simulation example, lagged effects affect the performance of the correlation measures and erroneous contemporaneous relationships between the variables are indicated.

Regarding the causality measures, PTENUE suggests the correct causal effects but additionally indicates the causal effect from $X_{1}$ to $X_{2}$. Based on the systems’s equations, by substituting $x_{1t}$ in the equation of $X_{2}t$, a lagged effect of $x_{1t-2}$ on $x_{2t}$ is obtained: $x_{2t}=\left(0.6x_{1t-2}+e_{1t}\right)+0.3x_{2t-1}+e_{2t}$. Therefore, the link $X_{1}$→$X_{2}$ is not erroneously found by the causality measures; a lagged relation emerges based on the equations of the system. RCGCI has similar performance to PTENUE; however, it also detects the indirect link $X_{1}$→$X_{3}$ with a low percentage (22%). PDC has again the worst performance overestimating the coupled pairs of variables.

Such a designed system favors the instantaneous causality measures, since both contemporaneous and causal effects exist. PMIME0 correctly identifies the contemporaneous dependence of $X_{1}$ and $X_{2}$ and the causal links, whereas also $X_{2}$ → $X_{1}$ is suggested (97%). As previously noted, moderately high percentages of significant effects are obtained also for non causal pairs of variables reaching 26% for $X_{4}$→$X_{5}$. PCMCI+ correctly infers the contemporaneous and causal dependencies; however, it seems to give high percentages of significant causal links in almost all directions.

## 6. Conclusions

In this paper, we have presented a brief review of the main connectivity measures currently used to infer the connectivity network of a examined complex system. Connectivity analysis is essential in different applications, such as in finance and neurophysiology. Nondirectional measures indicate the symmetric relationships of variables that evolve in synchrony, whereas directional measures infer the directions of the causal influences. The main limitations of the connectivity measures have been discussed in brief.

When studying the interdependencies of a system, connectivity may be inferred based on correlations or causality. However, selecting the proper methodology is still an open issue since the nature of real systems is in general unknown. This study investigated the efficacy of different connectivity measures for different simulated data, whereas complexity was further increased by considering non-normal noise terms in order to generate samples with skewed or/and non-symmetrical distributions. Simulation experiments were used to demonstrate the performance of the connectivity measures in three different scenarios, i.e., when data exhibit only contemporaneous dependencies, only directional causal effects, and finally both contemporaneous and temporal dependencies.

The main outcomes of the simulation study can be summarized to the following:

(a) Results suggest the sensitivity of correlation measures when temporal dependencies exist in the data. Correlation measures tend to erroneously indicate contemporaneous relations even though only lagged dependencies exist.
(b) Causality measures do not spuriously indicate causal effects when data present only contemporaneous dependencies. We should note here that the poor performance of PDC for systems 2 and 3 may be due to the fact that significant PDC values are reported comprehensively for all the examined frequencies. In real applications, usually specific frequency bands are selected according to the types of samples [211,212].
(c) Instantaneous causality measures handle contemporaneous and causal effects at the same time. Therefore, it seems to be highly promising for analyzing the connectivity structure of real data.
  Although both considered instantaneous causality measures seem to have potential and effectively infer the dependencies of most examined systems, they tend to give high percentages of significant causal effects for non-causal pairs of variables. This is a problem that explicitly reduces the effectiveness of the measures. The consideration of different values for the free parameters of the measures, such as the significance level or the number of neighbors for PMIME0, may improve the performance of the measures; however, here, only standard values of free parameters are used at all the examined systems for all causality measures. A possible optimization of the free parameters of the measures is out of the scopes of this work. However, the necessity of an automatic selection of standard free parameters of any connectivity measure in case of real applications should be pointed out.

The indicative simulation study highlights the limitations and advantages of the different connectivity measures. The outcomes of this study are suggesting the superiority of the causality measures over the correlation measures and the instantaneous causality measures. Correlation measures are highly affected by lagged directional relationships. Instantaneous causality measures, although promising, still need to be optimized to be effectively applied. At this point, we should note that correlation measures are effectively utilized long-term and are suitable for specific data types with possibly known topological features or characteristics, e.g., [213,214]. Further, their computation is extremely fast in contrast to the time-consuming estimation of most nonlinear causality measures.

Future studies aim to further investigate the above findings by testing additional scenarios regarding the samples and the nature of the dependencies, i.e., by considering samples with longer memory, samples that exhibit volatility clustering and samples of higher dimensions.

## Figures and Tables

**Table 1:** Non-directional connectivity measures.

Measure	Reference
Pearson product-moment correlation coefficient	[43]
Spearman rank correlation coefficient	[44]
Kendall’s rank correlation coefficient	[45]
Hoeffding’s test of independence	[46]
Biweight midcorrelation	[88]
Coefficient of determination	[48]
Distance correlation	[49,50]
Partial distance correlation	[51]
Yule’s Q	[52]
Yule’s Y	[53]
CANOVA	[9]
Randomized Dependence Coefficient	[56]
Mutual information	[65,66,67]
Nonlinear correlation information entropy	[64]
Entropy correlation coefficient	[68]
Entropy coefficient of determination	[69]
Maximal information coefficient	[70]
Partial maximal information coefficient	[71]
Coherence	[73]
Mean phase coherence	[12,79]
Phase locking value	[12,78]
Determinism	[83,84]

**Table 2:** Directional connectivity measures.

Measure	Reference
Granger causality	[1]
Conditional Granger causality	[111]
Partial Granger causality	[112]
Granger causality on radial basis functions	[113]
Granger causality on kernel functions	[114]
Granger causality on nonlinear autoregressive exogenous models	[115]
Baek and Brok test	[116]
Hiemstra and Jones test	[117]
Diks and Panchenko test	[118]
Nonlinear multivariate causality test of Hiemstra and Jones	[119]
Transfer entropy	[120]
Partial transfer entropy	[121,122]
Partial transfer entropy with nonuniform embedding	[123]
Mutual information on mixed embedding	[124]
Partial mutual information on mixed embedding	[125]
Low-dimensional approximation of transfer entropy	[126,127]
Nonlinear interdependence measures	[129,130,131,132,133,134]
(Conditional) extended Granger causality	[135]
PC algorithm	[138]
Fast Causal Inference	[140]
tsFCI	[145]
PCMCI	[146]
Geweke’s spectral Granger causality	[111]
Directed transfer function	[148]
Partial directed coherence	[149]
Direct directed transfer function	[150]
Generalized partial directed coherence	[151]
Phase Slope Index	[152]
Nonparametric partial directed coherence	[155]
DEKF-based Partial directed coherence	[156]
Nonlinear partial directed coherence	[157]
Extended Granger causality	[159]
Compensated transfer entropy	[168,169]
PMIME0	[171]
PCMCI+	[172]

**Table 3:** Percentage of significant correlations based on selected connectivity measures from 100 realizations of system 1. Rows drive the columns.

PPCor	1	2	3	4	5	PSpCor	1	2	3	4	5
**1**	-	6	6	15	5	**1**	-	5	4	87	3
**2**		-	100	11	100	**2**		-	100	5	100
**3**			-	1	3	**3**			-	6	100
**4**				-	10	**4**				-	5
**5**					-	**5**					-
**pdCor**	**1**	**2**	**3**	**4**	**5**	**MI**	**1**	**2**	**3**	**4**	**5**
**1**	-	4	4	98	1	**1**	-	9	4	31	4
**2**		-	100	1	100	**2**		-	100	3	100
**3**			-	5	100	**3**			-	2	100
**4**				-	4	**4**				-	1
**5**					-	**5**					-
**CGCI**	**1**	**2**	**3**	**4**	**5**	**RCGCI**	**1**	**2**	**3**	**4**	**5**
**1**	-	7	8	8	4	**1**	-	0	0	1	0
**2**	8	-	8	2	5	**2**	1	-	0	0	4
**3**	4	6	-	1	2	**3**	1	1	-	0	2
**4**	3	3	2	-	1	**4**	0	0	1	-	1
**5**	4	7	3	4	-	**5**	1	1	0	0	-
**PTENUE**	**1**	**2**	**3**	**4**	**5**	**PDC**	**1**	**2**	**3**	**4**	**5**
**1**	-	4	4	3	4	**1**	-	5	7	9	2
**2**	2	-	3	6	6	**2**	4	-	3	5	4
**3**	7	3	-	9	4	**3**	3	4	-	3	3
**4**	7	4	3	-	3	**4**	0	1	5	-	2
**5**	4	5	3	3	-	**5**	4	4	3	4	-
**Contemporaneous Effects**						**Causal Effects**					
**PMIME0**	**1**	**2**	**3**	**4**	**5**	**PMIME0**	**1**	**2**	**3**	**4**	**5**
**1**	-	2	5	42	4	**1**	-	5	28	22	6
**2**	5	-	100	5	100	**2**	19	-	16	15	15
**3**	3	98	-	3	3	**3**	19	2	-	25	4
**4**	41	3	7	-	3	**4**	18	2	15	-	4
**5**	3	100	2	1	-	**5**	13	11	7	11	-
**PCMCI+**	**1**	**2**	**3**	**4**	**5**	**PCMCI+**	**1**	**2**	**3**	**4**	**5**
**1**	-	5	7	89	2	**1**	-	12	7	7	10
**2**		-	100	2	100	**2**	9	-	12	8	9
**3**			-	5	3	**3**	6	3	-	11	9
**4**				-	7	**4**	11	7	8	-	11
**5**					-	**5**	9	7	8	8	-

**Table 4:** Percentage of significant correlations based on selected connectivity measures from 100 realizations of system 2. Rows drive the columns.

PPCor	1	2	3	4	5	PSpCor	1	2	3	4	5
**1**	-	10	100	17	7	**1**	-	12	100	17	8
**2**		-	16	9	6	**2**		-	10	8	5
**3**			-	9	3	**3**			-	8	3
**4**				-	100	**4**				-	100
**5**					-	**5**					-
**pdCor**	**1**	**2**	**3**	**4**	**5**	**MI**	**1**	**2**	**3**	**4**	**5**
**1**	-	72	100	28	10	**1**	-	12	99	8	5
**2**		-	96	9	7	**2**		-	13	5	5
**3**			-	11	4	**3**			-	8	4
**4**				-	100	**4**				-	23
**5**					-	**5**					-
**CGCI**	**1**	**2**	**3**	**4**	**5**	**RCGCI**	**1**	**2**	**3**	**4**	**5**
**1**	-	20	100	3	5	**1**	-	24	100	2	2
**2**	2	-	6	8	5	**2**	2	-	2	4	1
**3**	10	5	-	4	4	**3**	2	4	-	0	2
**4**	8	4	7	-	100	**4**	0	3	1	-	100
**5**	7	4	3	84	-	**5**	3	5	0	80	-
**PDC**	**1**	**2**	**3**	**4**	**5**	**PTENUE**	**1**	**2**	**3**	**4**	**5**
**1**	-	15	100	4	4	**1**	-	100	100	4	4
**2**	0	-	12	5	8	**2**	7	-	1	3	5
**3**	4	3	-	2	2	**3**	6	5	-	3	5
**4**	37	52	35	-	100	**4**	3	5	3	-	44
**5**	4	4	1	92	-	**5**	3	5	3	100	-
**Contemporaneous Effects**						**Causal Effects**					
**PMIME0**	**1**	**2**	**3**	**4**	**5**	**PMIME0**	**1**	**2**	**3**	**4**	**5**
**1**	-	7	0	3	7	**1**	-	100	100	8	13
**2**	5	-	2	5	4	**2**	12	-	5	11	13
**3**	7	6	-	3	5	**3**	12	14	-	6	16
**4**	5	5	0	-	13	**4**	15	11	6	-	74
**5**	7	2	3	3	-	**5**	15	16	8	100	-
**PCMCI+**	**1**	**2**	**3**	**4**	**5**	**PCMCI+**	**1**	**2**	**3**	**4**	**5**
**1**	-	6	7	3	4	**1**	-	100	100	19	10
**2**	6	-	4	5	7	**2**	21	-	41	14	8
**3**	7	4	-	3	5	**3**	32	21	-	13	13
**4**	3	5	3	-	4	**4**	13	7	14	-	88
**5**	4	7	5	4	-	**5**	12	12	10	100	-

**Table 5:** Percentage of significant correlations based on selected connectivity measures from 100 realizations of system 3. Rows drive the columns.

PPCor	1	2	3	4	5	PSpCor	1	2	3	4	5
**1**	-	100	100	2	38	**1**	-	100	100	4	30
**2**		-	100	2	11	**2**		-	100	5	10
**3**			-	100	100	**3**			-	100	100
**4**				-	43	**4**				-	41
**5**					-	**5**					-
**pdCor**	**1**	**2**	**3**	**4**	**5**	**MI**	**1**	**2**	**3**	**4**	**5**
**1**	-	100	0	0	0	**1**	-	100	24	2	10
**2**		-	100	0	1	**2**		-	89	6	19
**3**			-	100	100	**3**			-	53	96
**4**				-	91	**4**				-	8
**5**					-	**5**					-
**CGCI**	**1**	**2**	**3**	**4**	**5**	**RCGCI**	**1**	**2**	**3**	**4**	**5**
**1**	-	100	27	4	18	**1**	-	100	22	1	4
**2**	8	-	100	3	20	**2**	7	-	100	0	5
**3**	2	3	-	100	100	**3**	5	5	-	100	100
**4**	7	6	3	-	5	**4**	0	2	2	-	3
**5**	7	7	4	4	-	**5**	2	3	2	3	-
**PTENUE**	**1**	**2**	**3**	**4**	**5**	**PDC**	**1**	**2**	**3**	**4**	**5**
**1**	-	77	0	2	2	**1**	-	0	92	88	92
**2**	6	-	100	8	3	**2**	0	-	0	96	92
**3**	5	2	-	100	100	**3**	69	65	-	0	0
**4**	4	9	0	-	4	**4**	94	97	98	-	98
**5**	4	3	0	4	-	**5**	85	89	7	1	-
**Contemporaneous Effects**						**Causal Effects**					
**PMIME0**	**1**	**2**	**3**	**4**	**5**	**PMIME0**	**1**	**2**	**3**	**4**	**5**
**1**	-	100	0	8	10	**1**	-	0	13	21	18
**2**	100	-	1	5	5	**2**	97	-	100	18	18
**3**	0	0	-	7	5	**3**	0	0	-	100	100
**4**	0	0	0	-	2	**4**	0	0	4	-	26
**5**	0	0	0	3	-	**5**	0	1	1	22	-
**PCMCI+**	**1**	**2**	**3**	**4**	**5**	**PCMCI+**	**1**	**2**	**3**	**4**	**5**
**1**	-	100	2	7	5	**1**	-	39	2	36	33
**2**	100	-	1	8	3	**2**	32	-	100	49	30
**3**	2	1	-	2	3	**3**	26	27	-	72	100
**4**	7	8	2	-	6	**4**	17	21	61	-	36
**5**	5	3	3	6	-	**5**	23	35	52	33	-

## Data Availability

Not applicable.
